# Integrating HIV and substance use services: a systematic review

**DOI:** 10.7448/IAS.20.1.21585

**Published:** 2017-05-30

**Authors:** Victoria Haldane, Francisco Cervero-Liceras, Fiona LH Chuah, Suan Ee Ong, Georgina Murphy, Louise Sigfrid, Nicola Watt, Dina Balabanova, Sue Hogarth, Will Maimaris, Kent Buse, Peter Piot, Martin McKee, Pablo Perel, Helena Legido-Quigley

**Affiliations:** ^a^ Saw Swee Hock School of Public Health, National University of Singapore, Singapore; ^b^ Centre for Tropical Medicine and Global Health, Nuffield Dept. of Clinical Medicine, University of Oxford, Oxford, UK; ^c^ The Centre for Health and Social Change (ECOHOST), London School of Hygiene and Tropical Medicine, London, UK; ^d^ Department of Global Health & Development, London School of Hygiene and Tropical Medicine, London, UK; ^e^ London School of Hygiene and Tropical Medicine, London, UK; ^f^ London Borough of Waltham Forest, London, UK; ^g^ Haringey Council, London, UK; ^h^ UNAIDS, Geneva, Switzerland; ^i^ The World Heart Foundation, Geneva, Switzerland

**Keywords:** substance use, HIV, integration, public health, systematic review

## Abstract

**Introduction**: Substance use is an important risk factor for HIV, with both concentrated in certain vulnerable and marginalized populations. Although their management differs, there may be opportunities to integrate services for substance use and HIV. In this paper we systematically review evidence from studies that sought to integrate care for people living with HIV and substance use problems.

**Methods**: Studies were included if they evaluated service integration for substance use and HIV. We searched multiple databases from inception until October 2015. Articles were screened independently by two reviewers and assessed for risk of bias.

**Results and discussion**: 11,057 records were identified, with 7616 after removal of duplicates. After screening titles and abstracts, 51 met the inclusion criteria. Integration models were categorized by location (HIV, substance use and other facilities), level of integration from mirco (integrated care delivered to individuals) to macro (system level integrations) and degree of integration from least (screening and counselling only) to most (care for HIV, substance use and/or other illnesses at the same facility). Most reported descriptive or cohort studies; in four randomized control trials integrated activities improved patient outcomes. There is potential for integrating services at all facility types, including mobile health services. While services offering screening only can achieve synergies, there are benefits from delivering integrated treatment for HIV and substance use, including ease of referral to other mental health and social services.

**Conclusions**: Our review used a wide range of databases and conference archives to increase representation of papers from low- and middle-income countries. Limitations include the overrepresentation of studies from the United States, and the descriptive nature of the majority of papers. The evidence reviewed shows that greater integration offers important benefits in both patient and service outcomes but further research and outcome reporting is needed to better understand innovative and holistic care models at the complex intersection of substance use and HIV services.

## Introduction

People living with HIV often have other health-related problems, either as a consequence of immune suppression, treatment effects, shared risk factors, or a combination thereof [1–4]. This recognition has stimulated interest in the scope for integrating services traditionally provided separately where there is demonstrable patient population overlap. One such area is the intersection of substance use, especially involving exchange of blood, and the risk of HIV. Persons Who Inject Drugs (PWID) are 22 times more likely to acquire HIV than adults in the general population [5]. Indeed, despite decreasing global HIV transmission, PWID in many regions are experiencing marginally increasing HIV infections and worldwide an estimated 1.7 million PWIDs live with HIV [6–9]. PWID often face service exclusion, poor or fragmented care access and discrimination, which when coupled with punitive laws and reluctance to fund harm reduction programs, contribute to their growing HIV burden [9]. The overlap with substance use extends beyond injecting drug use, heavy episodic drinking of alcohol has been linked to risk behaviours such as unsafe sex and other drug use [10,11]. Although the clinical management of HIV and substance use differ, there is, theoretically, a case for exploring synergies in their management; however, caution and contextual sensitivity are warranted as there is no universal formula for integration, the overlap between conditions is only partial and in many societies both are stigmatizing in different ways [7].

The argument for service integration partially draws on general interest in health services integration, both at the policy level, where programs addressing single health problems can be brought together, and at the operational level, ensuring efficient use of scarce resources; thereby offering a way to improve access, responsiveness to patients’ needs, increase coverage, reduce inequalities, and improve health outcomes [12]. Integration is seen as particularly promising in high HIV burden and low resource settings, helping ensure complex health needs are addressed, building on care delivery and drug distribution system commonalities, facility sharing, and aligning funding mechanisms [13,14].

Calls to integrate HIV services with services for other health needs, such as the growing burden of non-communicable diseases (NCDs) in ageing populations living with HIV, have been relatively uncontentious. However, substance use presents a complicated situation as illicit drug policies are highly contested. Some governments advocate a criminal justice response and others a public health response. In 2016, governments attending the UN General Assembly Special Session (UNGASS on the World Drug Problem) reaffirmed their commitment to policies prohibiting the production, trafficking, possession and use of illicit drugs, nevertheless they have committed to provision of medical assisted therapy and clean injecting equipment [15,16]. The 2016 UN Political Declaration on HIV and AIDS, while acknowledging progress in health-related risk and harm reduction programs in some countries, noted the worldwide need for progress in reducing PWID HIV transmission and called for commitment to tailored prevention interventions [17]. This is echoed by the Johns Hopkins-Lancet Commission on Drug Policy and Health which calls for a harm reduction response, drawing on a growing body of evidence of effectiveness and embedded in a commitment to human rights [18].

While recognizing the need to consider how political factors may influence service integration, this review is part of a larger review that questioned when and in what circumstances are there benefits to bringing non-communicable disease and HIV services together. Consequently, in this paper we systematically review evidence from studies that sought to integrate care for people living with HIV and substance use problems.

There have been several global and regional level systematic reviews of HIV amongst PWID. However, these differed in scope and focus, examining the two conditions’ epidemiology [19–21] or treatment adherence determinants [22]. One assessed the impact of alcohol use disorders on HIV medication adherence, health care utilization and treatment outcomes while two, focusing on Africa, explored the association between alcohol use and HIV infection risk [23–25]. None, to our knowledge, has looked at integrating HIV and substance use services and programs.

## Methods

### Definitions

We drew on the integration definition proposed by Briggs, Atun and Legido-Quigley [26–28] whereby managerial or operational changes to health systems bring together inputs, delivery, management and organization of particular service functions with the aim of improving coverage access, quality, acceptability and/or (cost)-effectiveness. We included studies describing: service integration interventions; service delivery point integration; different levels of service delivery integration; process modifications; introduction of technologies aimed at aiding integration; and integration of management decisions [29,30]. We drew on an integration typology defining “service integration” as that integrating different clinical services through teams of multidisciplinary professionals and “clinical integration” as that integrating care into a single or coherent process with shared guidelines and protocols within and/or across professionals [31]. This typology differentiated integration at the macro level, as involving integration of the health system or major elements within it; the meso level, as involving organizations or professionals working together delivering integrated care to particular groups or populations, and the micro level, as involving providers delivering integrated person-centered care to individuals (Box 1) [31].

**Box 1.** Illustrative examples of integration typologies

	Micro	Meso	Macro
**Clinical integration** *Single or coherent process delivered by a provider who is equipped with shared guidelines and protocols*	A physician in a methadone maintenance program clinic also provides HIV counselling and testing services	A nurse working in a mobile health van for underserved populations is trained to provide both HIV counselling and DART with a clear referral pathway to a partnering HIV clinic, as well as substance use counselling with a clear referral pathway to a methadone maintenance clinic	N/A
**Service integration** *Teams providing different clinical services within the same organisation*	An HIV/AIDS clinic employs a substance use counsellor and physician and provides a methadone maintenance program for patients	A substance use program offering needle exchange sites hires HIV counsellors to provide outreach, education and referral to communities that utilize needle exchange services	N/A
**Systems integration** *Coordination between multi-location, multi-professional organisations to develop systems for the delivery of services for multiple conditions*	N/A	N/A	The HIV/AIDS Bureau, the Bureau of Drug Rehabilitation and the Bureau of Communicable Disease Control collaborate together and coordinate with their service providers, community members and stakeholders to enact policies and shared plans to decrease fragmentation of care for HIV/AIDS, substance use and hepatitis care

### Inclusion criteria

The review was conducted in accordance with PRISMA guidelines. We included quantitative and qualitative studies, as well as conference abstracts that reported the effects of health system level arrangements (service characteristics, interventions, policies or programmes) in different integrated care models for adults living with HIV and substance use. All studies describing or evaluating a managerial or organizational change to an existing health system that sought to increase integration of HIV and substance use services were included. For the present purposes we include within substance use harmful or hazardous psychoactive substances, alcohol and illicit drugs (morphine, heroin, tramadol, oxycodone and methadone) including non-medical use of prescription drugs. Companion papers (under review) discuss studies integrating mental health conditions or chronic medical conditions care with HIV.

We did not exclude reports based on study design; nor did we require outcome measures. There were no date or language restrictions. Studies that met inclusion criteria for this review were in English, French and Spanish language. No studies were excluded based on degree of assessed bias, although this was noted in interpreting the findings.

### Search strategy

The search strategy was developed with an information specialist for consistency with methods used in other health services integration systematic reviews [28,30]. The databases Global Health, Medline and Embase were searched from inception until October 2015. Key words (MeSH terms) and free text terms were developed for three themes: HIV, integration and chronic diseases and combined in the search strategy (Supplemental File 1). To ensure coverage of low- and middle-income countries, databases were searched using a simplified search strategy: Cochrane library, LILACs, Africa Wide, WHOLIS and abstracts from the International AIDS Society (IAS) Online Resource Library (2006–2015), the HIV Implementers meetings (2007–2012) and international conferences on NCDs including the 2014 Annual Meeting of the College on Problems of Drug Dependence and the 2015 Annual Scientific Meeting of the Research Society on Alcoholism among others.

### Search and retrieval of studies

Two reviewers independently reviewed the list generated by the electronic database search to identify relevant articles by title or title and abstract. Two reviewers independently assessed retrieved articles to determine whether they met inclusion criteria. Disagreements were resolved by discussion with a third reviewer.

### Data synthesis

Four reviewers independently extracted data from included studies using standardized forms developed to capture qualitative and quantitative study characteristics and results data. Data extraction or study interpretation differences were resolved by discussion and consensus. Data were extracted including information on: (1) study characteristics (study design, setting and sample size); (2) participants characteristics (age, gender, ethnicity and country of origin); (3) program or intervention integration activities; (4) results and outcome measure type (clinical, procedural and behavioural outcomes); and (5) integration activities’ advantages and disadvantages. Data were compared and disparities resolved. We conducted a narrative synthesis of study findings whereby after data extraction and using the extraction table we reviewed and sorted the studies into emergent groups. These groupings, which were based on location of the integration, then informed creation of the models.

### Risk of bias assessment

Four reviewers independently assessed risk of bias in studies that reported evaluative findings using the Cochrane risk of bias tool for randomized studies, a simple observational study proforma or an adapted checklist for qualitative studies [32]. We classified studies that had low risk of bias in all domains as low overall risk of bias. Studies that had high or unclear risk of bias in one or more domains were classified as overall high or unclear risk of bias.

## Results and discussion

**Figure 1.**
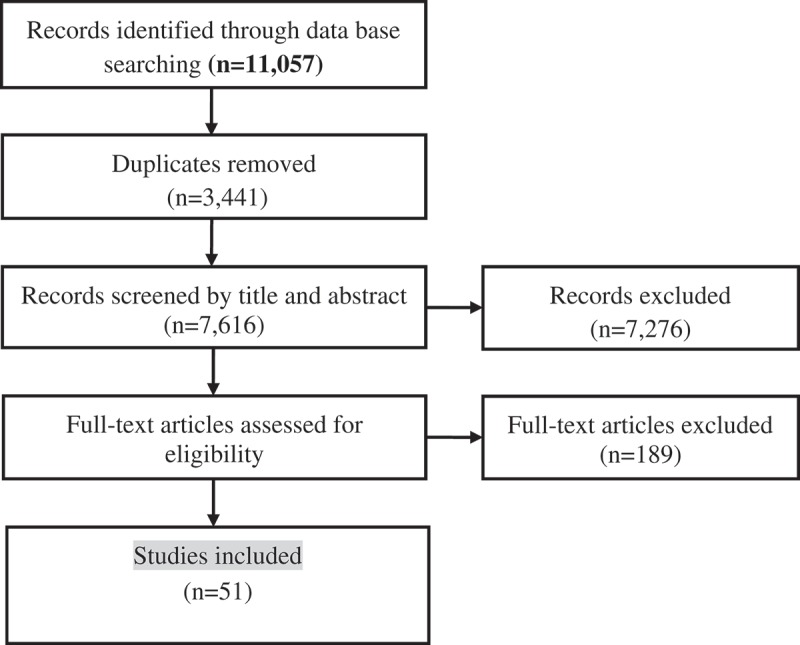
Study flow diagram.

Database searching identified 11,057 records, with 7616 remaining after duplicate removal, screened for inclusion by title and abstract, yielding 340 articles which were retrieved as full texts (Figure 1). Fifty-one articles met eligibility criteria, of which 47 were articles and four conference abstracts. We did not conduct a meta-analysis due to heterogeneity of study design, interventions, participants and outcomes, but instead present a descriptive summary of interventions, results and where available, outcomes.

### Models of integration

**Figure 2.**
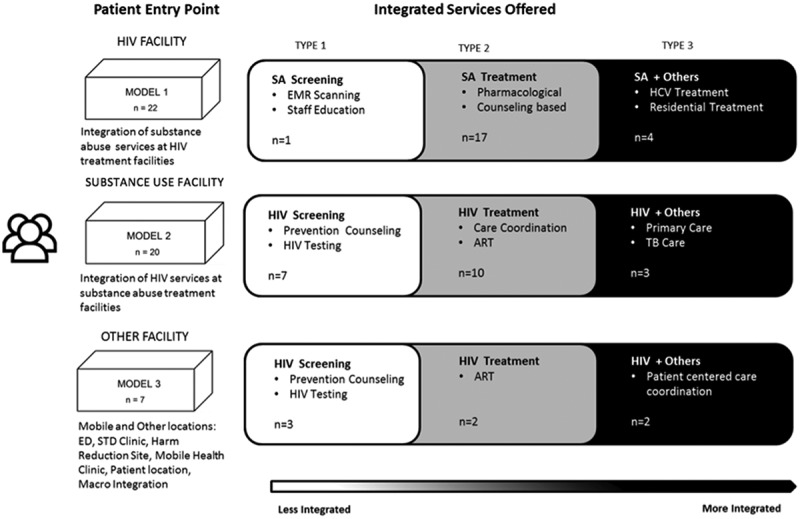
Models of integration of HIV and substance use services.

Integration models were defined by entry point at which a patient receives care; HIV facilities, substance use facilities and other facilities. We identified three integration types within these models (Figure 2). Type 1 integration includes facilities combining screening and counselling without further shared service provision. We consider screening-only to be least integrated, as it is less resource intensive and less complex than other forms of integration. Type 2 integration incorporates some treatment aspect, such as antiretroviral therapy (ART) provision in substance use facilities or substance use treatment in HIV facilities. The most integrated type combines substance use and HIV treatment, with other health care provision or social services; for example, drug and HIV treatment integration with harm reduction and palliative care [33,34].

### Characteristics of included studies

Fifty-one papers met the inclusion criteria; 22 papers described integration at HIV facilities, 20 at substance use facilities and seven at other facilities (sexually transmitted disease (STD) clinic, syringe access sites, emergency department and mobile health van). Two papers discussed patients’ perspectives on integration as a broader concept. Across sites, 11 involved screening activities, 29 treatment integration, and nine included services for other comorbidities. There were 18 descriptive studies; 14 cohort studies, seven qualitative studies, four RCT’s, one case-control, two mixed-methods and one cost analysis.

The majority of studies (*n* = 39) were conducted in the United States of America (USA) with two in Canada [33–74]. There were four European studies, two from Spain, one from Ireland and one from the Ukraine [75–78]. Two studies were from Africa, one each from Kenya and Tanzania [61,79]. There were four studies from Asia, one each from India, Indonesia, Taiwan and Vietnam [80–83]. The USA studies integrated care in all three facility types. Of the 41 studies from North America, 21 were located in HIV facilities. Elsewhere, only Kenya reported integration in an HIV facility [61]. Beyond North America, the majority of studies were in substance use facilities. Of ten non-North American studies, six were located in substance use facilities [75–77,79,80,82].

**Figure 3.**
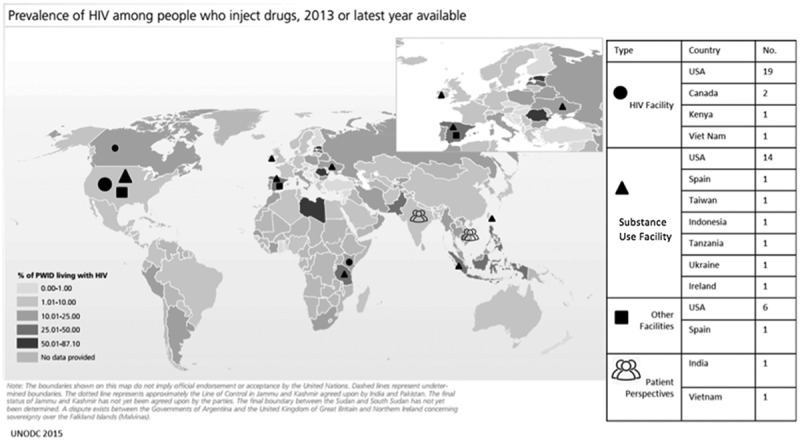
Map of integration by type and prevalence of HIV amongst persons who inject drugs.

Most integration examples were from areas with a low to moderate burden of HIV infection amongst people who inject drugs (PWID); no studies were from countries with over 50% of PWID living with HIV (Figure 3). Studies from Indonesia, Spain and Tanzania, which fall in the moderate range of PWID living with HIV (25.01–50%) involved HIV care integration into drug treatment programs [76,79,80]. Examples from countries with a lower range (10–25% PWID living with HIV) such as Canada, Kenya, and the Ukraine involved integration into HIV facilities, except for Ireland which integrated HIV services into addiction services [33,34,61,75].

Published examples were mostly from countries that allow access to both methadone (MMT) and buprenorphine maintenance treatment (BMT); only two studies were from areas that only offered MMT – Kenya and Vietnam [61,83]. India, which only offers BMT, was represented in one study [81].

Six papers offered an integration definition [45,55,57,63,69,75]. Two papers drew on Blount’s integrated service criteria where medical and behavioural elements are integrated into a treatment plan [57,63] and the remaining papers provided alternate definitions and criteria (Table 1).

**Table 1.** Definitions of integration from studies included in the review

Author	Definition of integration
Lombard et al. [57] and Proescholdbell et al. [63]	Blount (2003) criteria: integration of behavioural and medical elements into one treatment plan
Cheever et al. [45]	Integration as an ongoing process requiring assessment, planning, intervention and evaluation
Bachireddy [75]	Integration occurring on a spectrum with service co-location as “simple” integration and cross-disciplinary case management as more integrated
Sullivan [69]	Provider integration, where integration at the clinic level involves those services provided by different clinicians occurring at a single site, whereas individual integration is one where the treatment service is provided by the same clinician at a single site
Hoffman [55]	A formalized, collaborative process among services and systems with the goal of decreasing fragmentation of care and improving coordination

Integrations occurred at all system levels, but most (*n* = 30) described micro level service integration [33–41,44,47–49,51–53,57–59,61–63,66–71,76,77,79,80,82] (Table 2). Within HIV facilities, 15 studies explored micro level integration [33–37,48,49,52,61–63,67,69–71], one described meso level clinical integration [60] and five described macro level integration [45,50,57,73,74]. Similarly, within substance use facilities, the majority of studies were micro level integrations (*n* = 12) [40,41,44,47,51,58,59,66,68,76,77,79,80,82], with three meso level integrations [46,56,65]. In other facilities, three studies described micro level integration [38,39,53], two described meso level integration [42,43] and two described macro level integration [55,78].

**Table 2.** Overview of integration type by model from studies included in the review

Integration location	Integration type	*n* =
HIV facility	Micro-service integration [33–37,48,49,52,61–63,67,69–71]	15
	Macro-systems integration [33–37,48,49,52,61–63,67,69–71]	5
	Meso-clinical integration [60]	1
	ML^a^ [72]	1
Substance use facility	Micro-service Integration [41,44,47,51,58,59,66,68,76,77,79,80]	12
	Meso-clinical integration [46,56,65]	3
	ML [54,64,75]	3
	Micro-clinical integration [40,82]	2
Other facility	Micro-service integration [38,39,53]	3
	Meso-clinical integration [42,43]	2
	Macro-systems integration [55,78]	2
Patient perspectives	ML [81,83]	2

^a^ML describes those studies that explored multiple levels.

### Risk of bias assessment

We conducted risk of bias assessments for studies that evaluated service integration and reported outcome measures or qualitative results (Supplemental File 2). This included 23 studies; 13 cohort studies [36–40,42,62,63,66,68,76,79,80], four randomized control studies [35,52,61,69], two cross sectional studies [75,77], two qualitative studies [48,57], one case control study [59], and one cost analysis [67]. Fifteen had high risk of bias [36,37,39,40,48,62,63,68,69,75,77,79,80], six had moderate risk of bias [35,42,52,61,66,76] and two had low risk of bias [57,59].

### Model 1: integration of substance use services at an HIV facility

Twenty-two studies described integrated HIV and substance use care at HIV facilities. We define an HIV facility as an HIV clinic, HIV primary care clinic or other health care setting established to deliver HIV care. Within this model, the HIV facility offers different activities for substance use screening or treatment (Table 3).

**Table 3.** Summary of integrations provided at HIV facilities

Type	Activity	Author	*n* =
1. Substance use screening	Screening	O’Neill 2007 (USA)	1
2. Substance use treatment	BUP/NX	Cheever 2011 (USA), Turner 2005 (USA), Weiss 2011A (USA), Weiss 2011B (USA), Altice 2011 (USA), Finkelstein 2011 (USA), Schackman 2011 (USA), Egan 2014 (USA), Sullivan 2006 (USA), Draioni 2014 (USA)	10
	Counselling/MI	Hasin 2013 (USA), Aharanovich 2006 (USA), Aharanovich 2012 (USA) Lombard 2009 (USA), Proschold-Bell 2010 (USA), Parsons 2005 (USA), Papas 2011 (Kenya)	7
3. Substance use treatment + other treatments	HCV treatment	Taylor 2005 (USA), Taylor 2012 (USA)	2
	Residential Care	Krusi 2009 (Canada), McNeil 2014 (Canada)	2

### Type 1: substance use screening at an HIV facility

One study reported only substance use screening within an HIV clinic in the USA, where electronic medical record scanning identified patients misusing substances and clinic staff referred them for substance or mental health evaluation [60].

### Type 2: substance use treatment at an HIV facility

Eighteen papers incorporated substance use screening with treatment, including 10 studies from the USA that used buprenorphine naloxone (BUP/NX) for treatment. One sought to determine feasibility and efficacy of BUP/NX and psychosocial support in an HIV clinic, with no clear results [69].

Six papers reported on the American Buprenorphine HIV Evaluation and Support Initiative (BHIVES), which integrates BUP/NX into HIV care and involves a multisite evaluation with cross-site education and support [74]. Nine sites reported positive perceptions and described successful integration despite challenges with patient comorbidities and organizational barriers to program implementation, such as incorporating new procedures into established practice and limited adoption [73]. Patients had positive perceptions of BHIVES and attributed greater engagement with both HIV and substance use care to the integration [49]. Other studies reported positive perceptions of BUP/NX integration and the importance of counselling and social support in conjunction with pharmacological treatment [48].

Seven papers described counselling-focused interventions at HIV facilities. Telehealth motivational interviewing (MI) was used in three studies, two aiming to reduce drinking and one to reduce drug use; all reported positive patient outcomes [35,36,52]. One study explored an MI intervention, one a cognitive behavioral therapy (CBT) intervention and two described a combination MI and CBT approach provided to HIV patients at HIV facilities with positive results [61–63]. One study, conducted at HIV facilities in the USA, explored provider perceptions of clinic adaptations to integrate counselling; barriers including clinic size differences, space constraints, services offered, process design, professional autonomy and multidisciplinary team communication and collaboration [57].

### Type 3: substance use treatment + other treatments at an HIV facility

Four of 22 studies at HIV facilities described HIV care and substance use integration involving comorbidity treatment or a harm prevention approach. Two studies incorporated hepatitis C virus infection (HCV) care using a multidisciplinary model merging addiction, psychiatry, HIV and HCV treatment at a Centre for AIDS Research Coinfection Clinic [70,71]. Two studies from Canada explored an HIV day health and residential program providing medical care, counselling, social service referrals and a supervised injection program; staff reported greater trust and patient engagement, and patients felt supported in their safe injection habits, while social and structural determinants of health were better met, increasing treatment adherence [33,34].

### Model 2: integration of HIV services at substance use facilities

Twenty studies described care for HIV and substance use provided at substance use facilities (Table 4). Our definition of a substance use facility is a drug addiction/misuse/use centre or other healthcare setting primarily aiming to treat or mitigate the effects of drug addiction/misuse/use.

**Table 4.** Summary of integrations provided at substance use facilities

Type	Activity	Author	*n* =
1. HIV screening and prevention counselling	● Prevention counselling only	● Lee 2015 (Taiwan)	1
	● Screening and prevention counselling	● Seewald 2013 (USA), Kmeic 2012 (USA), Conners 2012 (USA), Henry 2010 (USA), Cartter 1990(USA), Gunn 2005 (USA)	6
2. HIV treatment	● Nurse led intensive care coordination	● Andersen 2003 (USA)	1
	● Pharmacological treatment	● Surah 2012 (Ireland), Sanchez 2012 (Spain), Cooperman 2007 (USA), Achmad 2009 (Indonesia), Berg 2009 (USA), Lucas 2004 (USA), Lucas 2007 (USA), Sorensen 2012 (USA), Tran & Bruce 2015 (Tanzania)	9
3. HIV treatment and other care services		● Bachireddy 2014 (Ukraine), Selwyn 1993 (USA), Rothman 2007 (USA)	3

### Type 1: HIV screening and prevention counselling at a substance use facility

Eight studies integrated HIV prevention counselling and screening at substance use facilities. One abstract described counselling without HIV screening in an MMT program in Taiwan [82]. Six papers described programs integrating prevention counselling with HIV screening activities. One paper provided an overview of state-wide integration of HIV services with MMT programs, indicating efficacy in testing linked to pre- and post-test counselling in substance use treatment facilities [44]. Another found evidence of efficacy of HIV and hepatitis B (HBV) counselling and testing, as well as HBV vaccination, in high risk patients in a non-residential drug-rehabilitation program [51]. Four of six studies from the USA focused on integrating rapid HIV testing into substance use treatment facilities. A study of patient acceptance in an ambulatory detoxification setting in the USA found rapid testing was acceptable to those with alcohol and opioid use [56]. Two studies from American Veterans Health centres that explored nurse led HIV rapid testing implementation found positive staff perceptions and readiness to provide HIV rapid testing. Reported barriers included increased staff workload, staff hesitancy performing medical procedures, and anxiety delivering HIV-positive results, as well as challenges linking patients to off-site HIV care [46,65].

### Type 2: HIV treatment at a substance use facility

Ten studies integrated HIV treatment at substance use facilities. One study from the USA described promising results from a nurse-led intensive care coordination protocol, where nurses accompanied participants for HIV treatment and facilitated integration of medical recommendations with substance use treatment [40].

Nine studies explored integrating pharmacological treatment (ART, HAART or DAART) into substance use treatment facilities. One prospective cohort study from Spain assessed HAART effectiveness at a drug use out-patient centre, where active drug users in the program achieved virological suppression rates on par with non-drug using matched subjects [76]. Seven studies explored HIV treatment in MMT programs. In Indonesia, an MMT program offered ART which improved HIV case detection and ART uptake [80]. Two studies in the USA offered HAART and directly observed therapy (DOT) treatment options to current and former opioid users in an MMT program, but with no clear results [41,47]. However, three studies from the USA explored integrating DAART into MMT programs in outpatient substance use treatment facilities with positive results for virological suppression; all faced participant drop out and poor adherence challenges [58,59,68]. In Tanzania, a study at an MMT that dispensed ARTs found evidence that methadone use enhanced client retention and linkage to care [79]. From a staff perspective, a study in Ireland of 30 addiction staff with HIV clinic links explored attitudes 6 months post integration showing positive staff perceptions of patients and of the integration [77].

### Type 3: HIV treatment + other conditions at a substance use facility

Three studies described integration of HIV and substance use treatment with management of other conditions, at substance use facilities. One study from the Ukraine assessed three integration models addressing substance use, HIV and TB, suggesting greater service integration increases ART access and improved patient quality of life [75]. In the USA, two studies explored primary care provision for HIV-infected drug users at substance use facilities with positive results; however, treatment paradigm differences between substance use and HIV care were reported as challenging to integration [64,66].

### Model 3: integration of HIV and substance use services at other facilities

Integration activities took place at five other facility types described in six papers, while one paper described a multi-location macro health systems integration of HIV, substance use and other services (Table 5).

**Table 5.** Summary of integrations provided at other facilities

Location	Type	Author	*n* =
Syringe access site	• HIV prevention counselling	• Burr 2014 (USA)	1
STD clinic	• HIV Testing	• Hennessey 2007 (USA)	1
Emergency dept	• HIV Testing & Prevention Counselling	• Bernstein 2012 (USA)	1
Mobile	• HIV Treatment	• Altice 2003 (USA), Altice 2004 (USA)	2
Multiple – patient centered	• HIV Care	• Tato 2000 (Spain)	1
Systems integration	• HIV + Substance Use + Mental Health + Hepatitis	• Hoffman 2004 (USA)	1

Two studies took place in clinical settings; one STD clinic and one emergency department. Both described HIV testing and prevention counselling amongst substance using patients and reported improved HIV testing uptake [42,53].

Three studies were located at harm reduction sites or mobile locations. One study from the USA explored a nurse-led health promotion program at syringe access sites and described efficacy in reaching large numbers of PWID [43]. Two studies described a mobile health clinic in the USA that provides HIV counselling, case management, drug treatment coordination, health status assessment, and medical care [38,39]. The papers reported improved virological results in patients receiving HAART and following DAART introduction, reported patient preference for mobile site treatment.

One paper described a patient-centered integration in Spain, offered jointly through the AIDS Patient Homecare Program (PADS) and Red Cross drug addiction program, delivered at patients’ preferred location[78]. PADS coordinated medical, substance use and comorbidity care and multiple social supports for patients and their families; reporting that substance use treatment, mainly through methadone, was important in patient success.

One paper described a systems level initiative in the USA, integrating service provision between government bureaus for HIV/AIDS, substance use and communicable diseases [55]. The main program output was a strategic plan offering joint assistance and provider training, coordination and information access, as well as joint procurement processes and contracting services.

### Patients’ perspectives

Two papers described patients’ perspectives on integrated HIV and substance use services, regardless of patient entry point. One cross-sectional study from Vietnam explored 510 MMT patient perspectives on integrated and decentralized MMT clinics and reported high preference for integration[83]. In India, a qualitative study explored HIV-positive PWID barriers to ART access and reported complex individual, social and system barriers while calling for supports built into existing treatment options [81].

### Measures of effectiveness of integration

32 studies evaluated one or more measures of effectiveness of the integrated intervention, program or model. We define *patient outcomes* as a change in patients’ health status, knowledge, behaviours or attitudes. We define *service delivery outcomes* as measures reflecting effectiveness in delivery of the service, program or integration, including staff perceptions (See Table 6).

**Table 6.** Types of outcome measures reported

	Types of outcomes reported (*n *= number of studies reporting outcomes)	
Models of integration	Patient outcomes	Service delivery outcomes
HIV facility (*n* = 22; *n* = 16 reported outcomes)	Identified with substance misuse disorder (1) ART uptake (1) ART adherence (2) BUP/NX adherence (1) CD4 count (2) Virological suppression (2) ASI score (2) Patient satisfaction and perspectives (5) Substance Use – Opioid (1) Substance Use – general (3) Number of Drinks per Day (3) Percentage drinking days (1) IVR Calls made (1) Confidence & temptation scores (1)	Referral to substance use or mental health evaluation (1) Median monthly provider encounters (1) Median monthly clinic costs per integrated care patient (1) Median monthly costs for BUP/NX (1) Staff satisfaction and perspectives (3)
Substance use facility (*n *= 20; *n* = 13 reported outcomes)	HIV testing acceptance (2) # rapid HIV tests performed (3) # newly diagnosed with HIV (1) Appointment adherence (1) Counselling adherence (1) Days to follow up (1) ART initiation (2) ART adherence (2) Hep B vaccination uptake (1) Receiving OST (1) ASI (1) Substance use (2) Global Well Being Scale (1) QHI composite scores (1) Perception of physical health, social functioning and mental health (1) Probability of CD4 screening (1) CD4 count (1) Virological suppression (3) Probability of virological response (1) Retention (2) Survival (1) Frequency of clinic visit (1)	Staff satisfaction and perspectives (2) Total annual cost per client served (1) Quality review (1)
Other Facility (*n* = 7; *n* = 3 reported outcomes)	Percentage of unprotected sex acts (1) Percentage of sex acts while high (1) Mean CD4 (1) Virological suppression (1) Entry to drug treatment (1) Percentage of ART doses taken (1)	Rates of adherence (1)
Patient Perspectives (*n* = 2; *n* = 0 reported outcomes)	N/A	N/A

One study reported long term outcomes of a macro integration from an organizational quality perspective [64]. Eight studies within HIV facilities were qualitative, reporting patient or staff perceptions of integration [33,34,48,49,57,73,81,83]. Two studies provided cost analyses, reporting on total integration cost as well as cost per client [51,67]. One study looked at three substance use disorder clinics and found that six months post rapid HIV testing implementation, acceptance rates and HIV testing were higher at the most integrated site [46]. Another study assessed different integration models, and reported Health Quality of Life (HRQoL), Quality Healthcare Index (QHI) composite scores and likelihood of patients’ receiving ART and opioid substitution therapy (OST) found patients receiving integrated care had significantly higher QHI scores compared to those receiving non-collocated services or harm reduction only (71.9% versus 54.8% versus 37.0%, *p* < 0.001); patients receiving integrated care were significantly more likely to receive ART (49.5% versus 19.2%, *p* < 0.001) [75].

Nineteen intervention studies were included [35–40,42,52,59,61–63,65,66,68,69,76,79,80]; 14 were cohort studies; two had a control group receiving usual care [76,80] one compared against seronegative patients [66] and 11 compared against baseline data [36–40,42,62,63,65,68,79]. One study was a case-control [59]. Four were RCTs [35,52,61,69], one compared a control (usual care group) with two intervention arms [52], one a usual care group against one intervention group [61]. Two papers compared two intervention groups against each other with no usual care control. All studies reported results pointing to positive outcomes from integration, but had high or moderate risk of bias; therefore while outcomes show promising results, the high risk of bias warrants caution in making assumptions on efficacy (Supplemental File 3).

### Discussion

This review explores different approaches to integrated HIV and substance use services based on patient entry points, synthesizing integration evidence at HIV, substance use and other facilities, as well as patient perspectives. The extent of integration varies, from combined screening activities to fully integrated screening, treatment and referrals for HIV, substance use and other comorbidities.

These models have advantages and disadvantages (Table 7). A positive finding is that across models the potential to increase HIV and substance use detection as well as provide structure, accountability and support for treatment adherence has been realized in some sites. Also, a single care provider may reduce the likelihood of drug interactions, while integrated services can facilitate communication across providers. When HIV, substance use and other conditions are managed together, enhancing continuity of care, there is evidence that acute episodes may be reduced, translating into reduced costs for patients [38,39]. However, some studies noted challenges to integration. For example, BHIVES achieved promising patient outcomes, but implementation barriers included higher costs, appropriate financing, workforce training, and challenges in combining differing clinical practices. Further, there is evidence that staff at substance use facilities may be hesitant to perform HIV testing, seen as a medical procedure, as well as in communicating positive HIV test results to patients. Integration also requires strong referral links to primary care, mental health and social services to address multiple and diverse patient needs [84]. A criminal justice approach to substance use has, in some regions, created legal barriers limiting pharmacological treatments and harm reduction activities [85]. Punitive approaches make it difficult to see the individual as a patient, preventing or delaying access to care [18,86].

**Table 7.** Summary of advantages and disadvantages reported in studies by integration model

	Model 1: HIV facilities	Model 2: substance use facilities	Model 3: other facilities
Potential advantages	*Substance use screening* Potential to increase substance use detection Minimal additional resources required *Substanceuse treatment* Provides structure, accountability, support and one touchpoint to support treatment adherence One treatment provider may reduce likelihood of negative drug interactions Facilitates communication across providers *Substance use + others* Potential to increase detection of HIV, substance use and other comorbidities Addresses social determinants through residential care or strong referrals	*HIV screening* Potential to increase HIV detection and patient awareness of HIV status Education platform Easy implementation of rapid testing *HIV treatment* Provides structure, accountability, support and one touchpoint to support treatment adherence One treatment provider may reduce likelihood of negative drug interactions Facilitates communication across providers *HIV + others* Potential to increase detection and treatment of HIV, substance use and other comorbidities Could reduce acute care episodes for patients reducing patient cost	*Clinical facilities* Potential to increase HIV detection and patient awareness of HIV status Easy implementation of rapid testing *Harm reduction and mobile facilities* Can access and serve marginalized groups Platform to build trust, teach safe injection practice and HIV risks and prevention Provides structure and one touch point to support treatment adherence Facilitates communication across providers Mobile clinic: perception of increased patient confidentiality – offers a suite of services, reduction of stigma *Patient led location* Robust case management and identification of comorbidities Could improve treatment adherence and monitoring Holistic view of patient and family needs and can link patient to other social services
Potential disadvantages	*Substance use screening* Requires staff training Loss to follow up *Substance use treatment* Legal barriers to provision of pharmacological treatment Requires strong linkages between pharmacological and counseling treatment Conceptual differences between HIV care and substance use care *Substance use + others* Policy and legal barriers to harm reduction approaches Cost of implementation	*HIV screening* Substance use staff hesitancy at performing HIV testing and giving positive test results *HIV Treatment* Requires links to primary care, mental health and social services Conceptual differences between HIV care and substance use care treatment *HIV + others* Cost of implementation	*Clinical facilities* Loss to follow up Requires staff training *Harm reduction, mobile and patient led facilities* Requires robust and dedicated outreach Requires additional staff, staff training and pharmacy coordination Loss to follow up due to patients’ social situation, incarceration, etc. Requires linkage to specialty care, acute care, mental health care and other social services Requires links to local police and other groups to map and understand the vulnerable population

We identified innovative approaches including integrated HIV and substance use care in mobile, community and residential settings. Such programs offer a people-centered approach allowing greater patient agency in managing substance use, HIV care and psychosocial supports. There is need for responsive, appropriate and convenient programs addressing the often-chaotic lives of people who use drugs [87] and these programs show promise reaching marginalized groups and provide a platform to build trust, educate, provide treatment and encourage adherence. Residential facilities have achieved success using robust harm reduction strategies coupled with housing, medical facilities and psychosocial support [33,34]. Other patient-centered programs utilized intensive nurse-led care to create coordinated protocols and foster provider-patient relationships [40]. These approaches require staff to be treatment advocates, use multidisciplinary approaches and have adequate access to not only mental health and clinical resources but social, transport and housing linkages [40,88,89].

Of note is the evidence describing patient perspectives of systems without integrated services, where patients encounter multiple family, social and system level barriers to care [81,83]. Patients reported fear of discrimination, unmet basic needs and unfriendly hospital environments and procedures, with inadequate counselling and perceived lack of confidentiality [81]. In contrast, studies describing fully integrated harm reduction approaches reported positive patient perceptions, especially of holistic care provision to address unmet social needs [33,34].

### Strengths and limitations

We used a wide range of databases and conference archives to increase paper representation from low- and middle-income countries. We also included studies published in languages other than English. However, studies were mostly from the USA, dealing with BUP/NX or the BHIVES initiative. Also, the review included many descriptive papers which, while providing insights into various integrated approaches, could not be used to infer effectiveness. In total 33 studies reported integration measures of effectiveness, four of which were RCTs in which methodological quality varied and overall had high or moderate risk of bias. Our review did not compare between integrated versus non-integrated services outcomes due to lack of literature. Further, due to the broad nature of the review we were unable to account for differences in treatment modalities for different types of substance use that may impact their ability to be effectively integrated into clinical care.

### Implications for research

Our review shows that research on integrated HIV and substance use care has focused on treatment approaches at the meso-level and small-scale clinical integration. There is need for further macro-level evaluations of systemic HIV and substance use service integration. Further, there is need to pursue research exploring meso-level integrations focusing on shared values and the tools necessary to overcome barriers obstructing links between HIV and substance use care.

Overall, there is need for longer term and more robust studies evaluating effectiveness. There is also a lack of long-term outcomes or relevant impacts on HIV and substance use, including reduced transmission rates and overall mortality. The longest follow up period reported in studies comparing outcomes was 12 months. This highlights the need for well-designed, robust studies with clear outcome indicators evaluating and comparing interventions. Also, further qualitative and mixed methods studies are necessary to better explore patient perspectives.

There is a particular need for research on integration approaches using model 3 whereby services are offered at diverse locations. While many studies described HIV testing initiatives, only one described a substance misuse screening initiative. Further research is required on sensitive approaches, such as community based or mobile service delivery, for specific needs of often vulnerable, transient and hard to reach populations. As the findings from studies of model 3 show, people living with HIV and substance use have diverse interactions with the health system and it is important to understand how to screen, refer or treat substance use in a variety of service provision settings.

Although our review included non-English papers and aimed for broad representation, only five studies were from low- or middle- income countries; one from Indonesia, one from Vietnam, one from India, one from Kenya and one from Tanzania, none from Latin America. There is need for studies from areas with a high burden of HIV and PWID, given their greater risk of HIV infection, and from different regions and health systems. However, as the lack of studies may be reflective of laws or policies in different regions placing prohibitions on substance use, it is important to address punitive approaches to substance use that may inhibit innovation and research on PWID.

## Conclusions

We identified three models integrating HIV and substance use; services in HIV facilities, substance use facilities and others. Benefits to integration are reported largely in terms of patient outcomes, including how integrated service can better enable patients to uptake and adhere to treatment; there are also demonstrable service outcomes including staff satisfaction with integrated approaches and easier referral to mental health and social services. Despite many countries pursuing a criminal justice approach, the UNAIDS 2016–2021 Strategy identifies the need to commit to service integration for those with drug dependency. The evidence reviewed here shows the need for innovative and holistic responses at the intersection of substance use and HIV services, especially the provision of integrated care at non-traditional sites and amongst vulnerable groups.

## Notes

To access the supplementary material to this article please see Supplementary Files under Article Tools online.
